# The Gut–Skin and Gut–Thyroid Axis in Autoimmunity: Roles of Dysbiosis, Microbial Metabolites, Immune Dysregulation, and Diet in Psoriasis and Hashimoto’s Thyroiditis

**DOI:** 10.3390/nu18101501

**Published:** 2026-05-08

**Authors:** Sabīna Ribačuka, Sabīne Upmale-Engela, Ieva Vaivode, Ilze Konrade, Māra Rone-Kupfere

**Affiliations:** 1 Faculty of Medicine, Riga Stradiņš University, LV-1007 Riga, Latvia; 040668@rsu.edu.lv; 2 Department of Internal Medicine, Riga Stradins University, LV-1007 Riga, Latvia; vaivode.ie@gmail.com (I.V.); ilze.konrade@rsu.lv (I.K.); 3 Department of Endocrinology, Riga East Clinical Hospital, Hipokrata Str. 2, LV-1038 Riga, Latvia; 4 Department of Dermatology and Venereology, Riga Stradiņš University, LV-1010 Riga, Latvia; mara.rone-kupfere@rsu.lv

**Keywords:** psoriasis, Hashimoto’s thyroiditis, gut microbiota, intestinal barrier dysfunction, gut–skin axis, gut–thyroid axis, bile acids, anti-inflammatory diet, Mediterranean diet

## Abstract

**Background/Objectives**: Psoriasis and Hashimoto’s thyroiditis are chronic immune-mediated disorders affecting distinct target organs but sharing overlapping pathogenic mechanisms, including gut dysbiosis, impaired intestinal barrier function, and systemic immune dysregulation. Growing evidence highlights the gut–skin and gut–thyroid axes as important interfaces linking microbial alterations to immune-mediated inflammation. This review aims to synthesize current knowledge on gut microbiota alterations in psoriasis and Hashimoto’s thyroiditis, with particular emphasis on intestinal permeability, immune pathways, and microbiome-derived metabolites. **Methods**: A narrative review of experimental and human observational studies was conducted to evaluate evidence on gut microbiota composition, intestinal barrier integrity, immune regulation, bile acid metabolism, and dietary influences in psoriasis and Hashimoto’s thyroiditis. The relevant literature examining mechanistic pathways and clinical associations was included. **Results**: Both conditions are associated with altered gut microbial composition, including reduced abundance of short-chain fatty acid–producing taxa, which may impair epithelial barrier integrity and promote systemic immune activation. Increased intestinal permeability and enhanced Th17-driven inflammatory responses are reported in both diseases. Recent studies suggest that dysregulated bile acid metabolism may influence intestinal permeability and immune balance along the gut–skin–thyroid axis, although direct clinical data remain limited. Dietary patterns, particularly anti-inflammatory and Mediterranean diets, are consistently associated with increased microbial diversity, improved metabolic profiles, and reduced systemic inflammation. However, most human evidence is observational. **Conclusions**: The gut microbiome represents a potential mechanistic link connecting diet, intestinal barrier function, immune regulation, and organ-specific autoimmunity in psoriasis and Hashimoto’s thyroiditis. While microbiome-targeted interventions show biological plausibility, well-designed, mechanistically informed randomized controlled trials are required to establish causality and clinical relevance.

## 1. Introduction

The intricate relationship between the gut microbiome and the immune system has gained increasing scientific attention, particularly in the context of chronic inflammatory diseases. Disruptions of the gut microbial ecosystem can trigger systemic immune responses that extend beyond the gastrointestinal tract and contribute to immune-mediated disorders, including psoriasis and Hashimoto’s thyroiditis (HT). Although these diseases affect distinct organs, they share overlapping inflammatory pathways, immune dysregulation, and microbiome-related mechanisms, highlighting the relevance of the gut–skin and gut–thyroid axes [1]. The shared and disease-specific immune pathways in psoriasis and Hashimoto’s thyroiditis are summarized in Table 1.

Psoriasis and HT are chronic immune-mediated conditions characterized by dysregulated T-cell responses, altered cytokine signaling, and associations with gut dysbiosis. In both diseases, enhanced Th1 and Th17 responses combined with reduced regulatory T-cell (Treg) function promote chronic inflammation [2,3,4]. Increased intestinal permeability (“leaky gut”) and microbial translocation, well documented in psoriasis and implicated more broadly in autoimmunity, may represent shared mechanisms amplifying systemic immune activation [2,5].

While shared pathways such as Th17-driven inflammation, microbial metabolite signaling, and intestinal barrier dysfunction provide a mechanistic link between psoriasis and Hashimoto’s thyroiditis, these diseases remain clinically and immunologically distinct entities. Therefore, the proposed gut–skin–thyroid axis should be interpreted as a conceptual framework highlighting shared upstream mechanisms rather than as a single unified pathophysiological model.

**Figure 1:**
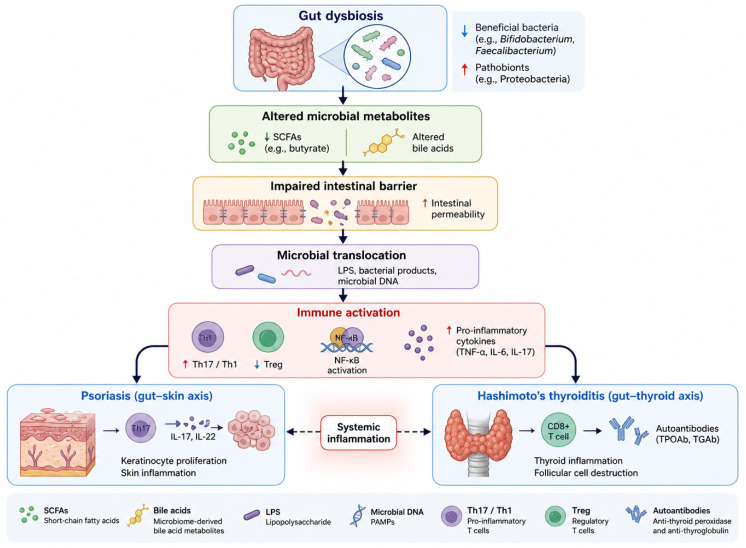
Pathogenesis pathway of psoriasis and Hashimoto thyroiditis.

Gut microbial metabolites play an important role in immune regulation and barrier integrity, linking gut dysbiosis with systemic inflammation relevant to both skin and thyroid disease [6,7,8]. In particular, short-chain fatty acids and bile acids represent key microbiome-derived signaling molecules whose immunomodulatory effects are discussed in detail below. The proposed mechanisms linking gut dysbiosis to immune dysregulation and the development of psoriasis and Hashimoto’s thyroiditis are illustrated in Figure 1.

Despite these shared features, the effector pathways and target tissues differ between two conditions. Psoriasis is a skin-directed inflammatory disorder characterized by excessive keratinocyte proliferation and Th17-driven cytokine production, whereas HT is an organ-specific autoimmune disease marked by lymphocytic thyroid infiltration, autoantibody production, and progressive follicular destruction [2,3,4,9,10].

This review aims to characterize gut microbiota alterations in psoriasis and Hashimoto’s thyroiditis and to assess the impact of diet on microbial composition, with particular emphasis on the gut–skin and gut–thyroid axes and their potential to modulate clinical outcomes.

## 2. Materials and Methods

A literature search was performed using PubMed/MEDLINE, Scopus, and Web of Science to identify relevant publications addressing the gut microbiome, intestinal barrier function, immune regulation, and autoimmune inflammatory diseases, with particular emphasis on psoriasis and Hashimoto’s thyroiditis.

The literature search included studies published between 2008 and 2025, with a primary focus on recent studies from the past five years (2020–2025). Earlier studies were included where relevant to provide mechanistic background.

Search terms included combinations of “*gut microbiota*”, “*gut dysbiosis*”, “*intestinal permeability*”, “*leaky gut*”, “*short-chain fatty acids*”, “*bile acids*”, “*Th17*”, “*regulatory T cells*”, “*gut–skin axis*”, “*gut–thyroid axis*”, “*psoriasis*”, “*Hashimoto’s thyroiditis*”, “*diet*”, “*anti-inflammatory diet*”, “*Mediterranean diet*”, and “*postbiotics*”. Original studies, experimental models, clinical trials, and relevant narrative and systematic reviews in English were included. Reference lists of selected articles were screened to identify additional relevant sources.

Given the heterogeneity of study designs and outcomes, findings were synthesized using a qualitative narrative approach, with a specific focus on the role of gut dysbiosis and intestinal barrier dysfunction in shaping immune responses, autoimmunity, and clinical manifestations of psoriasis and Hashimoto’s thyroiditis, as well as on the potential modulatory effects of diet and microbiome-targeted interventions.

## 3. Results

### 3.1. Intestinal Permeability (‘Leaky Gut’) in Autoimmune Disease

Disruption of the intestinal barrier together with alterations in gut microbiota composition has emerged as a unifying mechanism linking diverse autoimmune conditions. Across multiple studies, gut dysbiosis and increased intestinal permeability (“leaky gut”) appear not as secondary phenomena, but as early pathogenic events capable of initiating systemic immune disturbances. Impaired epithelial integrity, primarily due to alterations in tight-junction proteins, allows luminal antigens and microbial products to cross the mucosal barrier. This leads to the translocation of components such as lipopolysaccharides and bacterial DNA into the systemic circulation. This abnormal exposure activates innate and adaptive immune pathways and promotes pro-inflammatory signaling. It also skews T-cell differentiation, including enhanced Th17 activity, and contributes to the breakdown of immune tolerance [11,12,13].

It should also be noted that intestinal permeability remains a methodologically controversial concept. Commonly used biomarkers, such as zonulin, intestinal fatty acid-binding protein (I-FABP), and claudins, have limitations related to specificity, reproducibility, and variability across studies. Therefore, caution is required when interpreting intestinal permeability as a central mechanistic link in these conditions.

The literature consistently highlights the bidirectional relationship between dysbiosis and barrier dysfunction: microbial shifts can weaken tight-junction integrity, while increased permeability further reshapes the microbial environment, establishing a self-reinforcing cycle of inflammation. Environmental influences, including diet, stress, infections, and microbiota-driven metabolic changes, can exacerbate this cycle by impairing epithelial barrier regulation and amplifying immune dysregulation. These processes are now recognized as shared pathogenic features across several autoimmune diseases in both humans and murine models, supporting the concept that the gut–immune interface constitutes a critical checkpoint in autoimmunity [11,12].

The dysbiosis–barrier dysfunction axis is increasingly viewed as a modifiable target. Since the microbiome actively regulates tight-junction function, restoring microbial balance or strengthening epithelial barrier integrity may attenuate systemic immune activation and reduce autoimmune susceptibility. This paradigm suggests that therapeutic strategies aimed at modulating the gut ecosystem—through diet, probiotics, or barrier-supporting interventions—hold significant promise for preventing or mitigating autoimmune disease progression [11,12,13].

### 3.2. Short-Chain Fatty Acids as Mediators of Gut Barrier Integrity and Immune Regulation

Short-chain fatty acids (SCFAs), primarily acetate, propionate, and butyrate, are key microbial metabolites generated through fermentation of non-digestible dietary fibers. They constitute an essential link between gut microbiota composition, intestinal barrier integrity, and immune regulation [1,6]. SCFAs support epithelial homeostasis by enhancing tight-junction integrity, promoting epithelial renewal, and maintaining mucosal barrier function [1,6].

Beyond their barrier-stabilizing effects, SCFAs exert important immunomodulatory actions by regulating cytokine production and shaping T-cell differentiation. In particular, SCFAs promote regulatory T-cell (Treg) development and suppress excessive Th17-driven inflammatory responses, mechanisms central to immune tolerance and relevant to both psoriasis and Hashimoto’s thyroiditis [4,6].

Reduced abundance of SCFA-producing taxa has been consistently reported in both conditions. In psoriasis, decreased levels of key butyrate-producing species, including *Faecalibacterium prausnitzii* and *Akkermansia muciniphila*, are associated with impaired barrier integrity and increased systemic inflammation [2,3]. Similarly, in Hashimoto’s thyroiditis, reductions in beneficial SCFA-producing genera such as *Bifidobacterium* and *Lactobacillus* have been linked to increased intestinal permeability, immune dysregulation, and altered thyroid hormone metabolism [10,14,15,16,17].

Taken together, diminished SCFA production may represent a shared upstream mechanism linking gut dysbiosis to barrier dysfunction and immune imbalance in both skin and thyroid autoimmunity. Although current evidence is largely associative, SCFAs emerge as central mediators within the gut skin–thyroid axis and a biologically plausible target for diet- and microbiome-based interventions [1,6].

### 3.3. Bile Acids as Microbiota-Derived Mediators of Gut Barrier Integrity and Immune Regulation

Bile acids represent a critical but still underexplored signaling axis linking the gut microbiome with systemic immune regulation in chronic inflammatory and autoimmune diseases, including Hashimoto’s thyroiditis and psoriasis. Beyond their classical role in lipid digestion, bile acids function as bioactive molecules that influence intestinal barrier integrity, microbiome composition, and immune cell differentiation through both receptor-dependent and microbiota-mediated mechanisms.

Primary bile acids synthesized in the liver are converted into secondary bile acids by the gut microbiota through deconjugation, dehydroxylation, and epimerization reactions. Dysbiosis alters this microbial conversion process, leading to qualitative and quantitative changes in the bile acid pool [7]. In turn, bile acids exert potent antimicrobial effects, shaping microbiome composition and ecological balance. Unconjugated bile acids display particularly strong antibacterial activity, especially against Gram-positive bacteria [18], while distinct bile acids exhibit varying inhibitory properties. Alterations in bile acid composition can therefore drive further microbiota imbalance, potentially amplifying inflammatory states [19].

Accumulating experimental evidence demonstrates that bile acids play a significant role in regulating intestinal epithelial integrity. In vitro and in vivo studies indicate that specific bile acids can directly modulate tight junction structure and epithelial permeability. Micromolar concentrations of cholic acid, deoxycholic acid (DCA), and chenodeoxycholic acid have been shown to reduce transepithelial electrical resistance. They also increase paracellular flux through tight junction disruption [20]. Deoxycholic acid further induces dose-dependent epithelial barrier damage at physiologically relevant concentrations [21]. Consistently, positive correlations between intestinal permeability and secondary bile acid concentrations have been reported [22], while bile acid–specific and context-dependent effects on inflammation and epithelial permeability have also been demonstrated [23]. These observations are supported by comprehensive reviews concluding that bile acid dysregulation is closely associated with intestinal barrier dysfunction across multiple disease states [24].

Beyond barrier regulation, bile acids exert important immunomodulatory effects through activation of bile acid receptors, including the farnesoid X receptor (FXR) and Takeda G protein–coupled receptor 5 (TGR5). Reduced microbial conversion to secondary bile acids diminishes FXR/TGR5 signaling, which has been shown to impair regulatory T-cell (Treg) induction and favor Th17-skewed immune responses [19,24]. This shift toward Th17 dominance is particularly relevant given the established role of IL-23/Th17–IL-17 axis overactivation in both psoriasis and Hashimoto’s thyroiditis.

Both psoriasis and Hashimoto’s thyroiditis are increasingly recognized as Th17-driven immune-mediated disorders. In HT, traditionally viewed as a Th1-mediated disease, recent studies demonstrate increased numbers of Th17 cells and elevated IL-17 levels in thyroid tissue and peripheral blood [8,25]. Similarly, psoriasis is characterized by robust IL-17–dependent inflammation. Dysbiosis-associated alterations in bile acid metabolism may therefore represent a shared upstream contributor to immune dysregulation in both conditions.

Historical and clinical observations further support a link between bile acids and these diseases. Early studies suggested a contributory role of bile acids and endotoxins in psoriasis pathogenesis [26], while cross-sectional human data have demonstrated an inverse association between thyroid-stimulating hormone (TSH) levels and serum total bile acid concentrations, indicating potential endocrine–bile acid interactions relevant to thyroid function [27]. Nevertheless, despite emerging evidence of altered bile acid profiles in Hashimoto’s thyroiditis and psoriasis, direct clinical studies investigating causality and therapeutic relevance remain limited.

Although clinical translation remains insufficient, recent experimental data provide proof-of-concept for a therapeutic role of bile acids in inflammatory skin disease. In a murine model of psoriasiform dermatitis, administration of secondary bile acids attenuated skin inflammation, as reflected by reduced erythema, scaling, and epidermal thickening, concomitant with suppression of key psoriasis-associated cytokines, particularly IL-17A [28]. These effects were associated with decreased infiltration and activation of pro-inflammatory immune cells in the skin and reduced expression of IL-23/Th17 pathway components, indicating that bile acids can directly interfere with disease-relevant immune signaling through bile acid–dependent pathways. These findings support the concept that bile acids function as immunomodulatory molecules rather than solely metabolic byproducts and highlight bile acid receptors as potential therapeutic targets in psoriasis.

Overall, while preclinical data robustly demonstrate that bile acids influence intestinal permeability, microbiome composition, and Th17/Treg balance, no interventional trials have yet evaluated whether modulation of bile acid pools—through diet, microbiota-targeted therapies, or bile acid receptor agonists—can alter thyroid autoantibody titers (TPOAb, TgAb) or psoriasis disease severity. Thus, bile acids constitute a promising but insufficiently mapped mechanistic bridge within the gut–skin–thyroid axis, warranting further mechanistic and clinical investigation.

Although accumulating evidence supports a role for bile acids in modulating gut microbiota and immune responses, direct clinical intervention data remain limited. Therefore, these mechanisms should be interpreted as hypothesis-generating, and further clinical studies are required to establish their therapeutic relevance.

### 3.4. The Gut-Skin and Gut-Thyroid Axes in Immune-Mediated Inflammatory Diseases

**Figure 2:**
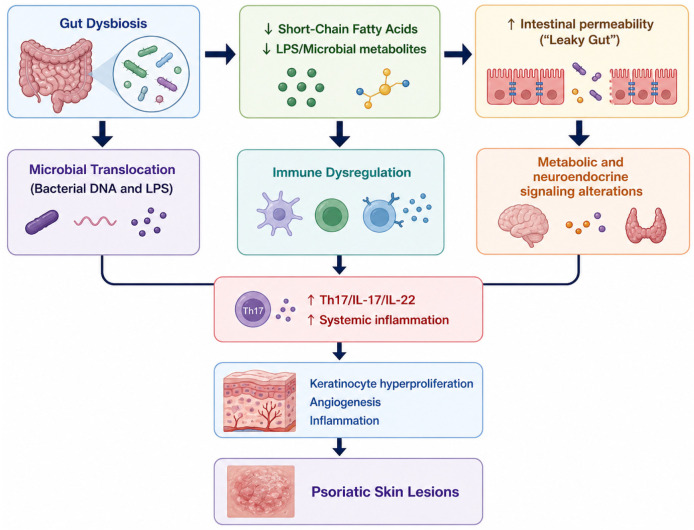
The gut–skin axis.

The human gastrointestinal tract harbors a complex microbial ecosystem essential for immune homeostasis [1]. Gut microbiota regulate epithelial barrier integrity and immune responses through the production of bioactive metabolites, particularly short-chain fatty acids (SCFAs) such as butyrate, which support tight-junction stability, epithelial renewal, and regulatory T-cell differentiation [1]. Disruption of microbial balance (gut dysbiosis) alters metabolic and immune pathways, leading to systemic inflammation and impaired skin function [3]. These mechanisms provide a mechanistic foundation for the gut–skin axis and its relevance to inflammatory skin diseases such as psoriasis [2,3]. A schematic overview of these mechanisms is presented in Figure 2.

**Figure 3:**
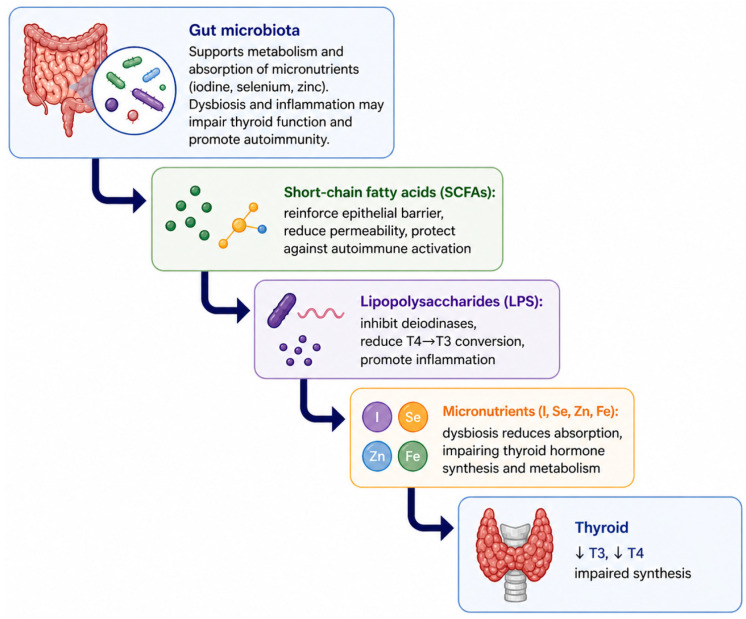
The gut–thyroid axis.

The gut–thyroid axis represents a bidirectional relationship between the intestinal microbiota and thyroid physiology. Gut dysbiosis disrupts immune tolerance, promotes systemic inflammation, and alters thyroid hormone metabolism through impaired microbial signaling and micronutrient availability [15,16]. The gut microbiota regulate the absorption of essential micronutrients, including iodine, selenium, zinc, and iron, which are required for thyroid hormone synthesis and conversion [15,16]. Microbiota-derived metabolites, particularly short-chain fatty acids (SCFAs), support intestinal barrier integrity and immune regulation. Other microbial metabolites, such as secondary bile acids, exert systemic endocrine effects by modulating type II iodothyronine deiodinase (D2), while lipopolysaccharides (LPS) from Gram-negative bacteria suppress both intestinal D2 and hepatic D1, thereby reducing peripheral activation of thyroid hormones [16]. Conversely, thyroid dysfunction can promote dysbiosis and increased intestinal permeability, establishing a self-reinforcing pathological loop linking gut imbalance to thyroid autoimmunity [29]. A schematic overview of these mechanisms is presented in Figure 3.

### 3.5. Gut Microbiota Alterations in Hashimoto’s Thyroiditis

Recent studies demonstrate a strong association between gut dysbiosis and autoimmune thyroid diseases (AITDs), including Hashimoto thyroiditis (HT) [14,15,16]. Disruption of the intestinal microbiota may impair epithelial barrier integrity, leading to increased intestinal permeability and facilitating the translocation of bacterial antigens. This process amplifies immune activation through mechanisms such as molecular mimicry, whereby microbial antigens share structural similarities with thyroid autoantigens and promote cross-reactive immune responses against thyroid follicular cells [17,30]. In parallel, dysbiosis has been associated with activation of Toll-like receptor 4–mediated pathways and shifts in helper T-cell profiles, contributing to autoimmune susceptibility [14].

Clinical and molecular studies consistently report distinct alterations in gut microbiota composition in HT patients. Culture-based, DGGE, and real-time PCR analyses have shown an overrepresentation of potentially pathogenic species, alongside marked reductions in beneficial genera such as *Bifidobacterium* and *Lactobacillus* [16]. High-throughput sequencing further confirms enrichment of taxa such as *Bacteroides*, *Escherichia–Shigella*, and *Parasutterella*, with concomitant decreases in *Prevotella* and *Dialister* when compared with healthy controls [16]. Quantitative analyses demonstrate significantly lower levels of *Bifidobacterium* in HT stool samples, while no significant differences were observed for other assessed taxa, including *Bacteroides*, *Clostridium coccoides*, *Clostridium leptum*, *Lactobacillus*, *Prevotella*, and *Roseburia*, although findings vary across studies [16]. These compositional changes and their associations with thyroid-related clinical parameters are summarized in Table 2 and Table 3.

There is no single dominant bacterial species consistently identified in Hashimoto’s thyroiditis; rather, the most recurrent pattern includes reduced *Bifidobacterium* and *Lactobacillus*, together with increased *Bacteroides*, *Escherichia*, and Proteobacteria.

These microbial shifts correlate with thyroid autoimmunity, showing positive associations between *Firmicutes* taxa and anti-TPO and anti-Tg autoantibody levels, and inverse correlations for *Bacteroidetes* taxa [17]. Additional correlations between specific genera and thyroid hormone parameters, including TSH and FT4, further underscore the functional relevance of these microbial patterns, as summarized in Table 2.

In contrast, several taxa appear to exert a protective role. Reduced abundance of *Alcaligenaceae*, *Pasteurellaceae/Pasteurellales*, *Peptococcaceae*, *Lachnospira*, and *Victivallis* has been associated with increased genetic susceptibility to thyroid autoimmunity, suggesting their involvement in immune regulation and maintenance of intestinal barrier integrity [14]. These observations highlight that gut dysbiosis in HT is not limited to pathogenic enrichment but also involves the loss of potentially protective microbial communities, as shown in Table 2.

Experimental and clinical intervention studies further support a functional role for microbiota modulation in thyroid regulation. In animal models, *Lactobacillus reuteri* supplementation increased free T4 concentrations, thyroid gland mass, and overall thyroid activity, potentially through interleukin-10–dependent activation of regulatory T cells [15]. Human studies similarly suggest that synbiotic supplementation may improve hypothyroid-related outcomes, including reductions in TSH levels, decreased levothyroxine requirements, and improved fatigue, although effects on thyroid autoantibody titers appear limited [15]. Together, these findings support the concept that gut dysbiosis and intestinal barrier dysfunction contribute to immune dysregulation, altered thyroid hormone metabolism, and disease progression in Hashimoto thyroiditis, as summarized in Table 2 and Table 3.

### 3.6. Gut Microbiota Alterations in Psoriasis

Gut dysbiosis has been implicated in several inflammatory skin diseases, most notably psoriasis [1,2]. In psoriasis, alterations in the gut microbiome may contribute to systemic inflammation through a self-perpetuating loop in which proinflammatory cytokines increase intestinal epithelial permeability, facilitating translocation of microbiome-derived products such as lipopolysaccharides and lipoteichoic acids into the systemic circulation [2]. Dysbiosis-related endotoxin–peptidoglycan superantigens may further promote autoimmune and autoinflammatory responses, supported by the detection of gut-derived bacterial antigens in psoriatic skin. Consistently, biomarkers of intestinal permeability, including claudin-3 and intestinal fatty acid–binding protein (I-FABP), are elevated in psoriasis patients, indicating gut barrier dysfunction [2].

Microbiome alterations in psoriasis are associated with activation of key inflammatory pathways, particularly the IL-23/IL-17/IL-22 axis, suppression of regulatory T cells, and sustained keratinocyte hyperproliferation [3]. In addition, NF-κB signaling (NFKB1) is a key transcriptional pathway involved in the pathogenesis of psoriasis, regulating inflammatory gene expression, keratinocyte proliferation, and cytokine production. Increased activation and phosphorylation of NF-κB have been observed in psoriatic lesions compared to non-lesional skin, and its activity is closely linked to pro-inflammatory mediators such as TNF-α, IL-1β, and IL-6, contributing to chronic skin inflammation [36]. Elevated systemic levels of proinflammatory mediators, including IFN-γ, IL-1β, IL-6, IL-12, and TNF, further link gut dysbiosis with systemic immune activation [1]. Similar inflammatory patterns are observed in other chronic inflammatory disorders, such as inflammatory bowel disease, supporting shared gut-driven mechanisms [3]. It should be noted that psoriasis is a heterogeneous disease encompassing multiple clinical phenotypes, and therefore microbiome alterations may not be uniformly applicable across all subtypes.

Psoriasis is characterized by alterations in Firmicutes/Bacteroidetes balance, with recurrent increases in *Faecalibacterium* and *Blautia* and decreases in *Bacteroidetes*, *Prevotella*, and *Alistipes*, although results vary across studies [1,2,3]. Additional taxa such as *Phascolarctobacterium* and *Dialister*, which belong to the Veillonellaceae family, have also been reported in psoriasis, although their roles remain less consistently defined across studies [5]. These changes may contribute to impaired barrier integrity, reduced SCFA availability, and systemic inflammation. Additional alterations at the family, genus, and species levels, as well as overgrowth of fungal and bacterial taxa associated with disease exacerbation, are summarized in Table 4 [2,9].

### 3.7. Dietary Modulation of the Gut Microbiome and Immune Regulation in Psoriasis and Hashimoto’s Thyroiditis

Diet represents the most significant modifiable environmental determinant of gut microbiota composition, with rapid and profound effects on microbial structure and function, while antibiotics, host genetics, and environmental factors also contribute to microbiome variability [37]. Different macronutrients influence microbial diversity, fermentation pathways, and the production of key metabolites, including short-chain fatty acids and bile acids, which are central to gut–skin and gut–thyroid axis signaling. An overview of how dietary fats, proteins, carbohydrates, and fiber modulate the gut microbiome and related inflammatory mechanisms is provided in Table 5 [1,7]. In this review, the primary focus is placed on dietary composition, particularly macronutrients and micronutrients, rather than total energy intake, as the analysis emphasizes microbiota-related mechanisms associated with specific dietary components.

While macronutrients primarily shape microbial composition and metabolic output, micronutrients and dietary factors exert additional immunomodulatory and barrier-related effects that may further influence disease-relevant pathways.

Dietary factors and micronutrient availability influence immune regulation, intestinal barrier integrity, and gut microbiota composition through interconnected pathways relevant to both Hashimoto’s thyroiditis and psoriasis [14]. An overview of key dietary micronutrients and dietary factors implicated in immune modulation, gut microbiome alterations, and disease-relevant mechanisms is presented in Table 6 [14].

Given the mechanistic focus of this review, particular emphasis is placed on Mediterranean and anti-inflammatory dietary patterns, as these have been most consistently associated with modulation of microbial metabolite production, bile acid signaling, and intestinal barrier integrity.

Anti-inflammatory and Mediterranean dietary patterns are of particular interest in this context. They share key compositional features, including high intake of plant-based foods, dietary fiber, and unsaturated fatty acids, particularly omega-3 fatty acids, along with restriction of processed foods and saturated fats. These factors are known to influence gut microbial diversity, short-chain fatty acid production, bile acid metabolism, and systemic immune responses. Although causal relationships cannot yet be firmly established, convergent observational and mechanistic evidence suggests that these dietary patterns may modulate shared pathogenic mechanisms relevant to both autoimmune thyroid disease and inflammatory skin disorders.

To facilitate a concise overview of the directionality and biological relevance of these diet-associated effects, Table 7 summarizes the impact of anti-inflammatory and Mediterranean dietary patterns on gut microbiota composition, intestinal barrier function, immune regulation, and disease-related outcomes in Hashimoto’s thyroiditis and psoriasis.

While anti-inflammatory and Mediterranean dietary patterns show promising associations with modulation of gut microbiota and inflammatory pathways, most of the available evidence is derived from observational studies. Therefore, these findings should not be interpreted as direct clinical recommendations, and further controlled intervention studies are required to establish their efficacy in patients with psoriasis and Hashimoto’s thyroiditis.

### 3.8. Postbiotics as Microbiome-Based Interventions

The term *postbiotic* derives from the Greek words *post* (“after”) and *bios* (“life”). Within the broader group of biotic-related terms-probiotics, prebiotics, synbiotics and postbiotics-all concepts revolve around microorganisms or their substrates. Postbiotics appropriately refer to preparations composed of non-living microbial cells or their components, including intact inactivated cells or structural fragments such as cell wall material. Many formulations also retain microbially produced substances, metabolites, proteins, or peptides, that may contribute to the biological effects attributed to postbiotics, although these components are not essential for classification. A preparation can be considered a postbiotic only when derived from well-defined microorganisms with known genomic sequences and produced through a standardized, reproducible process of biomass cultivation and inactivation [47].

Postbiotics have gained increasing attention in dermatology because they offer many of the functional advantages associated with probiotics while avoiding the practical and regulatory challenges posed by the use of viable microorganisms. Defined as non-viable microbial cells, their metabolic products, or structural components, postbiotics are recognized for their potential to stabilize the cutaneous microbiome and support skin health through anti-inflammatory and barrier-enhancing actions. Given the central role of the skin microbiome in maintaining cutaneous homeostasis—and the growing evidence implicating dysbiosis in acne, atopic dermatitis, psoriasis, and other inflammatory dermatoses—postbiotics represent an attractive strategy for modulating this ecosystem without the safety concerns linked to live bacterial administration. This has increased interest in both topical and oral postbiotic applications [48,49].

Postbiotics may promote skin barrier integrity by stimulating ceramide synthesis, improving hydration, and reducing transepidermal water loss, thereby reinforcing the stratum corneum and enhancing resistance to environmental stressors. In addition, many postbiotic-derived metabolites possess notable antioxidant and anti-inflammatory properties, offering protection against oxidative stress and attenuating inflammatory pathways that may contribute to the initiation or worsening of skin diseases [48,49].

Postbiotics may also influence skin immunity and structural cohesion. Certain bacterial lysates have demonstrated the ability to upregulate ceramide production, strengthen barrier integrity, and exert antimicrobial and immunomodulatory effects. Lysates of *Vitreoscilla filiformis*, for example, activate toll-like receptor signaling, stimulate neutrophil chemotaxis, and induce regulatory immune responses that collectively help reduce inflammation and restore cutaneous homeostasis [49,50].

Despite promising early findings, research on postbiotics remains in a relatively early stage. Much of the available evidence derives from in vitro experiments and animal models, and rigorously designed human clinical trials are limited. Key knowledge gaps persist regarding optimal dosing, long-term safety, their mechanisms of action within the complex skin microenvironment, and the most effective modes of topical or systemic delivery. Current reviews also highlight the need for clearer terminology, more robust safety assessments, and stronger regulatory oversight before postbiotics can be widely implemented in dermatological practice [49,50].

At present, no published studies have investigated the effects of postbiotics on thyroid function or thyroid-related diseases, including Hashimoto’s thyroiditis. Consequently, there is currently no scientific evidence supporting the use of postbiotics in the treatment or management of thyroid disorders.

## 4. Discussion

Accumulating evidence identifies the gut microbiome as a central regulator of immune homeostasis linking intestinal, cutaneous, and thyroid physiology. This review highlights that psoriasis and Hashimoto’s thyroiditis, despite distinct clinical manifestations, share overlapping pathogenic mechanisms driven by gut dysbiosis, impaired intestinal barrier function, and immune dysregulation. Although the gut–skin and gut–thyroid axes are often described separately, they likely represent interconnected components of a broader gut–immune–endocrine network. Shared mechanisms, including microbial metabolite signaling, intestinal barrier dysfunction, and Th17-driven immune responses, suggest potential crosstalk between these axes. These interactions may be modulated by dietary patterns, microbiome-targeted interventions, and factors influencing intestinal barrier integrity. Altered microbial composition and increased intestinal permeability facilitate systemic immune activation and contribute to the chronic inflammation characteristic of both conditions.

While individual gut–organ axes, such as the gut–skin and gut–thyroid axes, have been previously described, the integrated gut–skin–thyroid framework proposed in this review aims to highlight shared upstream mechanisms and potential interactions between these systems. Rather than representing a completely novel concept, this approach provides a more comprehensive perspective that may help identify common pathways and inform future research directions.

Microbial metabolites emerge as key mechanistic mediators within this gut–skin–thyroid axis. Reduced abundance of short-chain-fatty-acid-producing taxa may promote barrier dysfunction and immune imbalance, while dysregulated bile acid metabolism represents an important but still insufficiently explored pathway influencing intestinal permeability and Th17/Treg balance. The Th17/IL-17 axis constitutes a convergent immune pathway in both diseases, with gut-derived signals acting as upstream drivers of tissue-specific inflammation.

In addition to mechanistic insights, some studies report associations between gut microbiota alterations and clinical parameters. In psoriasis, microbiota changes have been linked to disease severity (e.g., PASI scores), while in Hashimoto’s thyroiditis, associations with thyroid function markers such as TSH and TPOAb have been observed. However, these findings are primarily observational and require further validation.

It is important to note that most evidence linking gut microbiota alterations, SCFA deficiency, and intestinal permeability to psoriasis and Hashimoto’s thyroiditis is derived from observational studies. Therefore, these findings should be interpreted as associative rather than causal, and further interventional and longitudinal studies are required to establish causality and therapeutic implications.

Despite growing associative evidence, important limitations remain. Most human studies are observational and heterogeneous, limiting causal inference and clinical translation. Direct evidence linking microbiome modulation to clinically meaningful outcomes, such as psoriasis severity or thyroid autoantibody levels, is still scarce. Consequently, well-designed randomized controlled trials are essential to determine whether dietary, probiotic, synbiotic, postbiotic, or other microbiome-targeted interventions can meaningfully alter disease course.

The variability and occasional inconsistency in reported microbiome alterations across studies may be explained by differences in study design, sequencing methodologies, population characteristics, medication use, and disease stage. These factors contribute to heterogeneity in the findings and limit the strength of conclusions. Therefore, current evidence should be interpreted with caution, and more standardized, large-scale studies are needed to establish consistent microbial patterns.

In conclusion, the gut microbiome represents a mechanistic bridge between diet, intestinal barrier integrity, immune regulation, and organ-specific autoimmunity in psoriasis and Hashimoto’s thyroiditis. While microbiome-targeted strategies hold therapeutic promise, their successful translation into clinical practice will require well-designed, mechanistically informed interventional studies.

## Figures and Tables

**Table 1:** Common and distinct immune pathways in psoriasis and Hashimoto thyroiditis.

Immunopathogenic Domain	Shared Mechanisms	Psoriasis	Hashimoto Thyroiditis
Disease nature	Chronic immune-mediated inflammatory diseases	Immune-mediated inflammatory skin disease	Organ-specific autoimmune thyroid disease
Primary target tissue	Peripheral organs affected by systemic immune activation	Skin (epidermis and dermis)	Thyroid gland
Initiating factors	Gut dysbiosis, barrier dysfunction, environmental triggers, diet	Dysbiosis-driven systemic inflammation	Dysbiosis, molecular mimicry, micronutrient imbalance
Intestinal barrier	Increased intestinal permeability (“leaky gut”) facilitating microbial translocation	Elevated gut permeability biomarkers (claudin-3, I-FABP); bacterial DNA detected in circulation	Increased permeability enabling antigen translocation and loss of immune tolerance
Innate immune activation	Activation of dendritic cells, macrophages, TLR-mediated signaling	Cutaneous dendritic cell and keratinocyte activation	Thyroid antigen presentation via APCs
Dominant adaptive immune response	Th1/Th17 with reduced regulatory control	Predominant Th17-driven response	Mixed Th1/Th17 response with cytotoxic CD8^+^ T-cell involvement
Key cytokine pathways	IL-17, IL-23, TNF-α, IFN-γ	IL-17A, IL-22, IL-23 → keratinocyte hyperproliferation	IFN-γ, TNF-α, IL-17 → thyroid follicular cell destruction
Regulatory T cells (Treg)	Impaired Treg differentiation and function	Reduced suppression of skin inflammation	Breakdown of thyroid immune tolerance
Role of Th17/IL-17 axis	Central convergent inflammatory pathway	Core pathogenic driver of disease	Increasingly recognized contributor to autoimmunity
Autoantibodies	Immune-mediated inflammation without dominant humoral autoimmunity	Not central to pathogenesis	Anti-TPO and anti-TG autoantibody production
Microbial metabolites -SCFAs	Reduced SCFA production leading to barrier dysfunction and immune dysregulation	Reduced SCFA production exacerbates skin inflammation	Reduced SCFAs impair immune tolerance and epithelial integrity
Microbial metabolites -bile acids	Dysregulated bile acid metabolism alters barrier integrity and Th17/Treg balance	Secondary bile acids modulate IL-17-dependent skin inflammation	Altered bile acid signaling affects immune balance and thyroid hormone metabolism

**Table 2:** Changes in gut microbiota associated with Hashimoto thyroiditis.

Study	Year	Microbiome Changes
Danailova et al., *Nutrients* (PMID 35563541) [15]	2022	↓ *Lactobacillus*, ↓ *Bifidobacterium*; impaired gut barrier integrity
Xiong et al., *Frontiers in Immunology* (PMC10865322) [14]	2024	↑ protective taxa: *Alcaligenaceae*, *Pasteurellaceae/Pasteurellales*, *Peptococcaceae*, *Lachnospira*, *Victivallis*
Dania Akeil et al., *Frontiers in Endocrinology* [28]	2023	↓ diversity; ↑ *Bacteroides*, ↑ *Escherichia*; ↓ *Lactobacillus*, ↓ *Bifidobacterium*
Fuya Zhao et al., *Thyroid* [17]	2018	↑ *Bacteroides*, ↑ *Prevotella*; ↓ *Faecalibacterium*, ↓ *Bifidobacterium*
H. M. Ishaq et al., *Biomedicine & Pharmacotherapy* [31]	2017	↓ *Firmicutes*; ↑ *Proteobacteria*; ↓ beneficial bacteria
Hong Zhao et al., *Polish Journal of Microbiology* [32]	2022	↓ *Bifidobacterium*, ↓ *Lactobacillus*; ↑ *Escherichia*, ↑ *Enterococcus*
I. Cornejo-Pareja et al., *Journal of Personalized Medicine* [33]	2020	↓ diversity; ↓ *Faecalibacterium*, ↓ *Roseburia*; ↑ *Bacteroides*
Leonardo C. F. Cayres et al., *Frontiers in Immunology* [16]	2021	↑ intestinal permeability; ↓ *Bifidobacterium*, ↓ *Lactobacillus*; ↑ *Enterobacteriaceae*
Miao Li et al., *Journal of Translational Medicine* [34]	2024	**Phylum:** ↑ *Proteobacteria*, ↑ *Actinobacteria*, ↑ *Bacteroidetes*, ↑ *Bacillota_A*, ↑ *Spirochaetota*, ↑ *Verrucomicrobia*; **Family:** ↓ *Verrucomicrobiaceae*, ↓ *Victivallaceae*, ↓ *Streptococcaceae*, ↓ *Rikenellaceae*; ↑ *Alcaligenaceae*, ↑ *Desulfovibrionaceae*, ↑ *Bacillaceae*; **Genus:** ↓ *Fecalibacterium*, ↓ *Bifidobacterium*, ↓ *Akkermansia*, ↓ *Coprococcus3*, ↓ *Butyrivibrio*; ↑ *Blautia*, ↑ *Roseburia*, ↑ *Ruminococcus torques group*, ↑ *Dorea*, ↑ *Bacteroides*, ↑ *Prevotella*, ↑ *Streptococcus*, ↑ *Alistipes*, ↑ *Escherichia–Shigella*, ↑ *Parasutterella*, ↑ *Intestinimonas*, ↑ *Turicibacter*, ↑ *Anaerostipes*, ↑ *Enterocloster citroniae*
N. Sawicka-Gutaj et al., *International Journal of Molecular Sciences* [35]	2022	↓ *Bifidobacterium*, ↓ *Lactobacillus*; ↑ *Bacteroides*, ↑ *Proteobacteria*
Simo Liu et al., *International Journal of Endocrinology* [10]	2020	Euthyroid HT: minor changes; Hypothyroid HT: ↓ *Bifidobacterium*, ↑ *Escherichia*, ↑ *Streptococcus*

Symbols: ↓ decreased; ↑ increased.

**Table 3:** Interactions Between Clinical Thyroid Parameters and Gut Microbiome Composition.

Clinical Parameter	Positively Correlated Taxa	Negatively Correlated Taxa	Study
TSH	*Clostridium coccoides*, *Clostridium coccoides-Eubacteria rectale*	*Bacteroidetes*, *Veillonella*, *Streptococcus*	Cayres et al., 2021 [16], Zhao et al., 2018 [17]
FT4	None reported	*Roseburia*	Cayres et al., 2021 [16]
TPOAb	*Bacteroidetes*, *Veillonella*, *Streptococcus*, *Bifidobacterium*, *Alistipes*, *Ruminococcaceae*, *Enterobacteriaceae*	*Faecalibacterium*	Cayres et al., 2021 [16], Zhao et al., 2018 [17]
Disease duration	*Clostridium coccoides*	None reported	Cayres et al., 2021 [16]

**Table 4:** Changes in gut microbiota associated with psoriasis.

Study (PMCID)	Year	Methodology	Microbiome Changes in Psoriasis
PMC7941898	2021	16S rRNA (QIIME, PICRUSt, LEfSe); Wilcoxon	↑ *Veillonellaceae*, ↑ *Ruminococcaceae*, ↑ *Faecalibacterium*, ↑ *Megamonas*; ↓ *Lachnospiraceae*
PMC7559734	2020	16S rRNA; LEfSe; OTU-level analysis	↑ *Faecalibacterium*; ↓ *Oscillibacter*, ↓ *Roseburia (Lachnospiraceae)*
PMC7177330	2020	16S rRNA; bacterial DNA detection in blood	↓ *Bacteroides*, ↑ *Faecalibacterium*; ↓ *Firmicutes*; ↓ *Actinobacteria* in psoriatic arthritis
PMC10094986	2023	16S rRNA; ANCOM; Mann–Whitney U	↑ *Blautia* (esp. *B. wexlerae*); ↓ *Parabacteroides distasonis*; *altered Akkermansia*, *S. aureus*, *S. pyogenes*, *C. albicans*
PMC8047475	2020	16S rRNA; Wilcoxon; COG & KEGG	↑ *Actinobacteria*, ↑ *Firmicutes*, ↑ *Verrucomicrobia*; ↓ *Bacteroidetes*, ↓ *Proteobacteria*, ↓ *Euryarchaeota*; ↑ *Faecalibacterium*, *Bacteroides*, *Bifidobacterium*, *Megamonas*, *Roseburia*; ↓ *Prevotella*, *Alistipes*, *Eubacterium*
PMC5799943	2018	16S rRNA;Weighted UniFrac; LEfSe; PMI	↑ *Firmicutes*, ↓ *Bacteroidetes*; ↑ *Faecalibacterium*, *Blautia*; ↓ *Bacteroides*, *Paraprevotella*
PMC10933079	2023	16S rRNA; LEfSe; metagenomeSeq; PSO rat model	↑ *Firmicutes*, ↑ *Actinobacteriota*; ↓ *Bacteroidota*; ↑ *Faecalibacterium*, *Megamonas*, *Prevotella*, *Escherichia-Shigella*; Rats: ↑ *Lachnospiraceae*, *Bacteroides*, *Roseburia*; ↓ *Alloprevotella*, *Muribaculaceae*, *Alistipes*
PMC10002560	2022	16S rRNA; metagenomic analysis	↑ *Firmicutes*, ↑ *Actinobacteriota*, ↑ *Akkermansia*; ↓ *Bacteroidetes*; ↑ *Blautia*, *Faecalibacterium*; ↓ *Prevotella*, *Alistipes*

Symbols: ↓ decreased; ↑ increased.

**Table 5:** Effects of dietary macronutrients on gut microbiome composition, microbial metabolites, and disease-relevant pathways in Hashimoto’s thyroiditis and psoriasis.

Dietary Component	Effect on Gut Microbiome	Key Microbial/Metabolic Mechanisms (SCFAs/Bile Acids)	Relevance to Psoriasis and HT
**Saturated fatty acids**	Pro-inflammatory microbial shifts	Altered bile acid metabolism favoring inflammatory signaling	May exacerbate gut dysbiosis and immune dysregulation linked to autoimmune disease [38]
**Unsaturated fatty acids (Omega-3)**	Less detrimental effects on microbial composition	More favorable bile acid signaling	Potentially lower inflammatory impact compared with saturated fats [38]
**Dietary protein (excess intake)**	↑ Proteolytic fermentation	↑ Harmful metabolites (IS, TMAO, p-cresyl sulfate, ammonia, phenols, indoles, H_2_S)	Associated with systemic inflammation and immune activation relevant to psoriasis and HT [1,38]
**Dietary protein (collagen, whey, pea protein)**	↑ *Lactobacillus*, *Bifidobacterium*; ↓ pathogenic taxa	↑ SCFA production; improved mucosal barrier	May support barrier integrity and reduce inflammatory pathways in autoimmune skin and thyroid disease [1]
**Dietary fiber (high intake)**	↑ Beneficial taxa (*Bifidobacterium*, *Lactobacillus*, *Faecalibacterium*)	↑ SCFA production (acetate, propionate, butyrate);	Supports barrier integrity, regulatory immune responses, and attenuation of systemic inflammation relevant to both psoriasis and HT

Symbols: ↓ decreased; ↑ increased.

**Table 6:** Effects of dietary micronutrients and dietary factors on immune regulation, gut microbiota, and disease-relevant pathways in Hashimoto’s thyroiditis and psoriasis.

Dietary Factor	Key Effects on Immune Regulation and Barrier Function	Microbiome-Related Mechanisms	Relevance to HT and Psoriasis
**Vitamin D**	Modulates T-cell responses; ↑ IL-10; reduces inflammatory signaling	Indirect effects via immune–microbiome interactions	↓ TPO-Ab and improved TSH in HT; improved cutaneous immune balance in psoriasis [4,12]
**Iron**	Essential for thyroid hormone synthesis; deficiency increases inflammatory stress	May indirectly alter microbial composition through metabolic imbalance	Iron deficiency impairs TPO activity in HT and may exacerbate systemic inflammation affecting psoriasis [4]
**Selenium**	Supports antioxidant defense and thyroid hormone metabolism	Influences redox balance affecting microbial–host interactions	↓ Thyroid autoantibodies in HT; may reduce oxidative stress–driven inflammation in psoriasis [4]
**Antioxidant vitamins (A, C, E, B-group)**	Reduce oxidative stress; regulate immune responses	Support epithelial integrity and microbial homeostasis	May contribute to normalization of inflammatory responses in both HT and psoriasis [12]
**Polyphenols (resveratrol)**	Suppress NF-κB signaling; attenuate Th17-driven inflammation	Promote beneficial microbial taxa and anti-inflammatory metabolites	Reduce inflammatory signaling in psoriasis; support immune regulation relevant to HT [12]
**Carotenoids**	Regulate immune signaling via retinoic acid (RAR/RXR pathways)	Indirect modulation of immune–microbiome crosstalk	Support immune homeostasis in inflammatory and autoimmune disease [12]
**Alcohol**	Disrupts immune regulation; ↑ CRP	Alters microbiota composition and barrier integrity	Associated with psoriasis exacerbations and impaired immune balance relevant to HT [39]

Symbols: ↓ decreased; ↑ increased.

**Table 7:** Effects of anti-inflammatory and Mediterranean diets in Hashimoto’s thyroiditis and psoriasis.

Dietary Pattern	Main Effects
Anti-inflammatory diet	↑ Gut microbiota diversity and health-associated taxa [40,41] ↑ SCFA-producing bacteria and SCFA availability [40,41] ↑ Antioxidant defenses and microbial homeostasis [15,42] ↓ Pro-inflammatory microbial pathways and chronic immune activation [40,41,42,43] ↓ Oxidative stress and inflammatory mediator expression in psoriasis [42] ↓ Circulating anti-TPO and anti-Tg antibody levels (observational associations) [42] ↓ Disease triggers (refined sugars, processed foods, saturated fats, alcohol) and psoriasis exacerbations [42]
Mediterranean diet	↑ Beneficial gut microbiota and SCFA-producing taxa [40,41] ↑ Butyrate production and intestinal epithelial integrity [40,41] ↑ Tight-junction stability and regulatory immune responses [40,41] ↑ Favorable bile acid profiles supporting immune regulation [40,42] ↓ Th17-driven inflammatory signaling relevant to psoriasis and HT [15,42] ↓ Oxidative stress and systemic inflammation [41] ↓ Thyroid autoimmunity prevalence and thyroid autoantibody levels (observational data) [42] ↓ Psoriasis disease activity with higher adherence; ↑ activity with poor adherence and processed food intake [44,45,46]

Symbols: ↓ decreased; ↑ increased.

## Data Availability

Not applicable.

## Abbreviations

The following abbreviations are used in this manuscript:

AITDs	Autoimmune thyroid diseases
APCs	Antigen-presenting cells
D1	Type I iodothyronine deiodinase
D2	Type II iodothyronine deiodinase
FXR	Farnesoid X receptor
HT	Hashimoto’s thyroiditis
I-FABP	Intestinal fatty acid-binding protein
IFN-γ	Interferon gamma
IL	Interleukin
LPS	Lipopolysaccharides
NF-κB	Nuclear factor kappa B
SCFAs	Short-chain fatty acids
TGR5	Takeda G protein-coupled receptor 5
Th	T helper cell
TLR	Toll-like receptor
TNF-α	Tumor necrosis factor alpha
TPOAb	Thyroid peroxidase antibodies
Treg	Regulatory T cells
TSH	Thyroid-stimulating hormone

## References

[1] Mahmud M.R., Akter S., Tamanna S.K., Mazumder L., Esti I.Z., Banerjee S., Akter S., Hasan M.R., Acharjee M., Hossain M.S. (2022). Impact of Gut Microbiome on Skin Health: Gut-Skin Axis Observed through the Lenses of Therapeutics and Skin Diseases. Gut Microbes.

[2] Polak K., Bergler-Czop B., Szczepanek M., Wojciechowska K., Frątczak A., Kiss N. (2021). Psoriasis and Gut Microbiome—Current State of Art. Int. J. Mol. Sci..

[3] Olejniczak-Staruch I., Ciążyńska M., Sobolewska-Sztychny D., Narbutt J., Skibińska M., Lesiak A. (2021). Alterations of the Skin and Gut Microbiome in Psoriasis and Psoriatic Arthritis. Int. J. Mol. Sci..

[4] Kaur J., Jialal I. Hashimoto Thyroiditis. https://www.ncbi.nlm.nih.gov/books/NBK459262/.

[5] Zhang X., Shi L., Sun T., Guo K., Geng S. (2021). Dysbiosis of Gut Microbiota and Its Correlation with Dysregulation of Cytokines in Psoriasis Patients. BMC Microbiol..

[6] Madaan T., Doan K., Hartman A., Gherardini D., Ventrola A., Zhang Y., Kotagiri N. (2024). Advances in Microbiome-Based Therapeutics for Dermatological Disorders: Current Insights and Future Directions. Exp. Dermatol..

[7] Guzior D.V., Quinn R.A. (2021). Review: Microbial Transformations of Human Bile Acids. Microbiome.

[8] Jadali Z., Esfahanian F., Ghelich R., Rashidian H. (2017). Increased Levels of Serum Interleukin-17 in Patients with Hashimoto’s Thyroiditis. Indian J. Endocrinol. Metab..

[9] Thye A.Y.-K., Bah Y.-R., Law J.W.-F., Tan L.T.-H., He Y.-W., Wong S.-H., Thurairajasingam S., Chan K.-G., Lee L.-H., Letchumanan V. (2022). Gut–Skin Axis: Unravelling the Connection between the Gut Microbiome and Psoriasis. Biomedicines.

[10] Liu S., An Y., Cao B., Sun R., Ke J., Zhao D. (2020). The Composition of Gut Microbiota in Patients Bearing Hashimoto’s Thyroiditis with Euthyroidism and Hypothyroidism. Int. J. Endocrinol..

[11] Christovich A., Luo X.M. (2022). Gut Microbiota, Leaky Gut, and Autoimmune Diseases. Front. Immunol..

[12] Paray B.A., Albeshr M.F., Jan A.T., Rather I.A. (2020). Leaky Gut and Autoimmunity: An Intricate Balance in Individuals Health and the Diseased State. Int. J. Mol. Sci..

[13] Li B., Selmi C., Tang R., Gershwin M.E., Ma X. (2018). The Microbiome and Autoimmunity: A Paradigm from the Gut–Liver Axis. Cell. Mol. Immunol..

[14] Xiong Y., Zhu X., Luo Q. (2024). Causal Relationship between Gut Microbiota and Autoimmune Thyroiditis: A Mendelian Study. Heliyon.

[15] Danailova Y., Velikova T., Nikolaev G., Mitova Z., Shinkov A., Gagov H., Konakchieva R. (2022). Nutritional Management of Thyroiditis of Hashimoto. Int. J. Mol. Sci..

[16] Cayres L.C.D.F., De Salis L.V.V., Rodrigues G.S.P., Van Helvoort Lengert A., Biondi A.P.C., Sargentini L.D.B., Brisotti J.L., Gomes E., De Oliveira G.L.V. (2021). Detection of Alterations in the Gut Microbiota and Intestinal Permeability in Patients with Hashimoto Thyroiditis. Front. Immunol..

[17] Zhao F., Feng J., Li J., Zhao L., Liu Y., Chen H., Jin Y., Zhu B., Wei Y. (2018). Alterations of the Gut Microbiota in Hashimoto’s Thyroiditis Patients. Thyroid.

[18] Tian Y., Gui W., Koo I., Smith P.B., Allman E.L., Nichols R.G., Rimal B., Cai J., Liu Q., Patterson A.D. (2020). The Microbiome Modulating Activity of Bile Acids. Gut Microbes.

[19] An C., Chon H., Ku W., Eom S., Seok M., Kim S., Lee J., Kim D., Lee S., Koo H. (2022). Bile Acids: Major Regulator of the Gut Microbiome. Microorganisms.

[20] Raimondi F., Santoro P., Barone M.V., Pappacoda S., Barretta M.L., Nanayakkara M., Apicella C., Capasso L., Paludetto R. (2008). Bile Acids Modulate Tight Junction Structure and Barrier Function of Caco-2 Monolayers via EGFR Activation. Am. J. Physiol.-Gastrointest. Liver Physiol..

[21] Stenman L.K., Holma R., Eggert A., Korpela R. (2012). A Novel Mechanism for Gut Barrier Dysfunction by Dietary Fat: Epithelial Disruption by Hydrophobic Bile Acids. Am. J. Physiol.-Gastrointest. Liver Physiol..

[22] Murakami Y., Tanabe S., Suzuki T. (2015). High-fat Diet-induced Intestinal Hyperpermeability Is Associated with Increased Bile Acids in the Large Intestine of Mice. J. Food Sci..

[23] Calzadilla N., Comiskey S.M., Dudeja P.K., Saksena S., Gill R.K., Alrefai W.A. (2022). Bile Acids as Inflammatory Mediators and Modulators of Intestinal Permeability. Front. Immunol..

[24] Shi L., Jin L., Huang W. (2023). Bile Acids, Intestinal Barrier Dysfunction, and Related Diseases. Cells.

[25] The Role of Th17 Cells and IL-17 in Hashimoto’s Thyroiditis: Review. https://pubmed.ncbi.nlm.nih.gov/38710521/.

[26] Role of Bile Acids and Endotoxins in the Pathogenesis and Therapy of Psoriasis. https://pubmed.ncbi.nlm.nih.gov/10827473/.

[27] Song Y., Zhao M., Zhang H., Zhang X., Zhao J., Xu J., Gao L. (2015). Thyroid-Stimulating Hormone Levels Are Inversely Associated With Serum Total Bile Acid Levels: A Cross-Sectional Study. Endocr. Pract..

[28] Alkader D.A.A., Asadi N., Solangi U., Singh R., Rasuli S.F., Farooq M.J., Raheela F.N.U., Waseem R., Gilani S.M., Abbas K. (2023). Exploring the Role of Gut Microbiota in Autoimmune Thyroid Disorders: A Systematic Review and Meta-Analysis. Front. Endocrinol..

[29] Knezevic J., Starchl C., Berisha A.T., Amrein K. (2020). Thyroid-Gut-Axis: How Does the Microbiota Influence Thyroid Function?. Nutrients.

[30] Pei X.-Q., Wang W.-H., Gao Y.-H., Zhang T.-X., Liu J.-Y., Zhao Z.-D., Zhang H.-W. (2024). Role of Immune Cells in Mediating the Effect of Gut Microbiota on Hashimoto’s Thyroiditis: A 2-Sample Mendelian Randomization Study. Front. Microbiol..

[31] Ishaq H.M., Mohammad I.S., Guo H., Shahzad M., Hou Y.J., Ma C., Naseem Z., Wu X., Shi P., Xu J. (2017). Molecular Estimation of Alteration in Intestinal Microbial Composition in Hashimoto’s Thyroiditis Patients. Biomed. Pharmacother..

[32] Zhao H., Yuan L., Zhu D., Sun B., Du J., Wang J. (2022). Alterations and Mechanism of Gut Microbiota in Graves’ Disease and Hashimoto’s Thyroiditis. Pol. J. Microbiol..

[33] Cornejo-Pareja I., Ruiz-Limón P., Gómez-Pérez A.M., Molina-Vega M., Moreno-Indias I., Tinahones F.J. (2020). Differential Microbial Pattern Description in Subjects with Autoimmune-Based Thyroid Diseases: A Pilot Study. J. Pers. Med..

[34] Li M., Chen K., Chen Y., Zhang L., Cui Y., Xiao F., Liu Z., Zhang W., Jiang J., Zhou Q. (2024). Integrative Analysis of Gut Microbiome and Host Transcriptome Reveal Novel Molecular Signatures in Hashimoto’s Thyroiditis. J. Transl. Med..

[35] Sawicka-Gutaj N., Gruszczyński D., Zawalna N., Nijakowski K., Muller I., Karpiński T., Salvi M., Ruchała M. (2022). Microbiota Alterations in Patients with Autoimmune Thyroid Diseases: A Systematic Review. Int. J. Mol. Sci..

[36] Sieminska I., Pieniawska M., Grzywa T.M. (2024). The Immunology of Psoriasis—Current Concepts in Pathogenesis. Clin. Rev. Allergy Immunol..

[37] Makki K., Deehan E.C., Walter J., Bäckhed F. (2018). The Impact of Dietary Fiber on Gut Microbiota in Host Health and Disease. Cell Host Microbe.

[38] Schoonakker M.P., Van Peet P.G., Van Den Burg E.L., Numans M.E., Ducarmon Q.R., Pijl H., Wiese M. (2024). Impact of Dietary Carbohydrate, Fat or Protein Restriction on the Human Gut Microbiome: A Systematic Review. Nutr. Res. Rev..

[39] Svanström C., Lonne-Rahm S.-B., Nordlind K. (2019). Psoriasis and Alcohol. Psoriasis Targets Ther..

[40] Bagheri S., Zolghadri S., Stanek A. (2022). Beneficial Effects of Anti-Inflammatory Diet in Modulating Gut Microbiota and Controlling Obesity. Nutrients.

[41] Yu X., Pu H., Voss M. (2024). Overview of Anti-Inflammatory Diets and Their Promising Effects on Non-Communicable Diseases. Br. J. Nutr..

[42] Mikulska A.A., Karaźniewicz-Łada M., Filipowicz D., Ruchała M., Główka F.K. (2022). Metabolic Characteristics of Hashimoto’s Thyroiditis Patients and the Role of Microelements and Diet in the Disease Management—An Overview. Int. J. Mol. Sci..

[43] Garbicz J., Całyniuk B., Górski M., Buczkowska M., Piecuch M., Kulik A., Rozentryt P. (2021). Nutritional Therapy in Persons Suffering from Psoriasis. Nutrients.

[44] Zhang M., Fan S., Hong S., Sun X., Zhou Y., Liu L., Wang J., Wang C., Lin N., Xiao X. (2024). Epidemiology of Lipid Disturbances in Psoriasis: An Analysis of Trends from 2006 to 2023. Diabetes Metab. Syndr. Clin. Res. Rev..

[45] Duchnik E., Kruk J., Tuchowska A., Marchlewicz M. (2023). The Impact of Diet and Physical Activity on Psoriasis: A Narrative Review of the Current Evidence. Nutrients.

[46] Katsimbri P., Korakas E., Kountouri A., Ikonomidis I., Tsougos E., Vlachos D., Papadavid E., Raptis A., Lambadiari V. (2021). The Effect of Antioxidant and Anti-Inflammatory Capacity of Diet on Psoriasis and Psoriatic Arthritis Phenotype: Nutrition as Therapeutic Tool?. Antioxidants.

[47] Vinderola G., Sanders M.E., Salminen S. (2022). The Concept of Postbiotics. Foods.

[48] De Almeida C.V., Antiga E., Lulli M. (2023). Oral and Topical Probiotics and Postbiotics in Skincare and Dermatological Therapy: A Concise Review. Microorganisms.

[49] Zdybel K., Śliwka A., Polak-Berecka M., Polak P., Waśko A. (2025). Postbiotics Formulation and Therapeutic Effect in Inflammation: A Systematic Review. Nutrients.

[50] Shi Z., Wu X., Wu C.-Y., Singh S.P., Law T., Yamada D., Huynh M., Liakos W., Yang G., Farber J.M. (2021). Bile Acids Improve Psoriasiform Dermatitis through Inhibition of IL-17A Expression and CCL20-CCR6–Mediated Trafficking of T Cells. J. Investig. Dermatol..

